# Changing expression patterns of TonB-dependent transporters suggest shifts in polysaccharide consumption over the course of a spring phytoplankton bloom

**DOI:** 10.1038/s41396-021-00928-8

**Published:** 2021-03-01

**Authors:** T. Ben Francis, Daniel Bartosik, Thomas Sura, Andreas Sichert, Jan-Hendrik Hehemann, Stephanie Markert, Thomas Schweder, Bernhard M. Fuchs, Hanno Teeling, Rudolf I. Amann, Dörte Becher

**Affiliations:** 1 Max Planck Institute for Marine Microbiology, Bremen, Germany; 2 Institute for Pharmacy, University of Greifswald, Greifswald, Germany; 3 Institute of Marine Biotechnology, Greifswald, Germany; 4 Institute for Microbiology, University of Greifswald, Greifswald, Germany; 5 Center for Marine Environmental Sciences (MARUM), Bremen, Germany

**Keywords:** Water microbiology, Molecular ecology, Proteomics

## Abstract

Algal blooms produce large quantities of organic matter that is subsequently remineralised by bacterial heterotrophs. Polysaccharide is a primary component of algal biomass. It has been hypothesised that individual bacterial heterotrophic niches during algal blooms are in part determined by the available polysaccharide substrates present. Measurement of the expression of TonB-dependent transporters, often specific for polysaccharide uptake, might serve as a proxy for assessing bacterial polysaccharide consumption over time. To investigate this, we present here high-resolution metaproteomic and metagenomic datasets from bacterioplankton of the 2016 spring phytoplankton bloom at Helgoland island in the southern North Sea, and expression profiles of TonB-dependent transporters during the bloom, which demonstrate the importance of both the *Gammaproteobacteria* and the *Bacteroidetes* as degraders of algal polysaccharide. TonB-dependent transporters were the most highly expressed protein class, split approximately evenly between the *Gammaproteobacteria* and *Bacteroidetes*, and totalling on average 16.7% of all detected proteins during the bloom. About 93% of these were predicted to take up organic matter, and for about 12% of the TonB-dependent transporters, we predicted a specific target polysaccharide class. Most significantly, we observed a change in substrate specificities of the expressed transporters over time, which was not reflected in the corresponding metagenomic data. From this, we conclude that algal cell wall-related compounds containing fucose, mannose, and xylose were mostly utilised in later bloom stages, whereas glucose-based algal and bacterial storage molecules including laminarin, glycogen, and starch were used throughout. Quantification of transporters could therefore be key for understanding marine carbon cycling.

## Introduction

Seasonal phytoplankton blooms are known to be responsible for a substantial proportion of total annual atmospheric carbon fixation [1–3]. Many studies have found characteristic prokaryotic clades, primarily members of the *Bacteroidetes*, *Gammaproteobacteria*, and *Alphaproteobacteria*, transiently achieve high abundance while consuming and thus remineralising algal biomass [2, 4–7]. Not only are the higher-level taxa dominant, but individual genera and species have been shown to be present and abundant year to year [5, 6, 8–11].

The *Bacteroidetes* are often considered specialists at degrading high molecular weight (HMW) substrates such as protein and polysaccharide [12–17]. Characteristically, their genomes encode homologues of the starch utilisation system (Sus), specifically the SusC-like TonB-dependent transporter (TBDT), and the SusD-like accessory substrate-binding protein [18, 19]. SusCD-like transporter complexes can provide a substrate-specific ‘selfish’ uptake system for poly- and oligosaccharides (e.g. [20]) that permits sequestration of organic matter in the periplasm (e.g. [21]) for subsequent breakdown into monosaccharides.

TBDTs are also widely distributed among other Gram-negative bacteria such as *Gammaproteobacteria* (e.g. [14, 22, 23]) or *Cyanobacteria* (e.g. [24]), and are known to transport bulky compounds such as iron-siderophores, nickel complexes, vitamin B12, and oligosaccharides (e.g. [25–30]), for reviews see [31, 32]. Several proteins are involved in TonB-dependent transport, including the inner membrane ExbB and ExbD proteins and the linking TonB-protein itself. For our purposes, we use TBDT to refer to only the initial substrate binding and transporting outer membrane pore proteins. SusC-like proteins form a smaller, *Bacteroidetes*-specific radiation within the overall universe of TBDTs, thus all SusC-like proteins are TBDTs, but not all TBDTs are SusC-like proteins. Only the *Bacteroidetes* are known to have evolved the characteristic accessory SusD-like substrate-binding protein typically found in conjunction with their SusC-like proteins. It has recently been proposed that SusCD-like proteins may transport not only polysaccharides, but also other macromolecules such as peptides (e.g. [18]) or nucleic acids [33].

In *Bacteroidetes*, *susC-* and *susD*-like genes are frequently located in tandem within operon/regulon-like genetic structures known as polysaccharide utilisation loci (PULs). These PULs contain the transporter genes and genes for carbohydrate-active enzymes (CAZymes)—in particular glycoside hydrolases, polysaccharide lyases, and carbohydrate esterases—required for cleaving specific larger oligo- and polysaccharides [14, 34–36]. Thus, the CAZyme gene content of a PUL is often useful for predicting its approximate polysaccharide specificity [10, 15, 33, 37]. Recent work has used this approach to show relatively limited PUL diversity during spring phytoplankton blooms, suggesting only a few polysaccharide types predominate [10]. Meanwhile, metaproteome studies have found that TBDTs are among the most abundant proteins in the nutrient-rich oceans [6, 7, 38–42], implying that active uptake via TonB-dependent transport plays a central role in marine surface water ecosystems.

In this study, we present one of the largest environmental metaproteomic datasets to date, comprising more than 27 000 detected protein groups. These data are temporally resolved, as they span six sampling points over the 2 months from March to May during the 2016 spring phytoplankton bloom at the island of Helgoland in the southern North Sea. The depth achieved in each sample permitted better identification of broad trends in protein abundance and thus functional potential across the bacterioplankton community. In addition, we produced nine accompanying metagenomic datasets. After assembly and binning of these to generate metagenome assembled genomes (MAGs), we identified 1261 expressed TBDTs during the bloom belonging to many species of *Bacteroidetes* and *Gammaproteobacteria*, the majority of which could only be assigned a putative non-specific dissolved organic matter (DOM) uptake function. Nevertheless, it was possible to determine that expression of transporters with predicted polysaccharide substrates changed with bloom phase, suggesting that abundance of specific polysaccharide transporters may be useful as a proxy for understanding polysaccharide utilisation in these environments.

## Materials and methods

### Environmental sampling

Samples were collected at the island of Helgoland in the North Sea at the long-term research station ‘Kabeltonne’ (54°11′17.88″N, 7°54′0″E) during the 2016 spring bloom. Six metaproteome samples were collected on March 17 and 31, April 19, and May 3, 12, and 17, while metagenome samples were collected on March 16, 21, and 31, April 12, 19, and 26, and May 2, 12, and 17. Sampling procedures for both metaproteomics and metagenomics have been described previously [5, 6]. Briefly, water for metagenomic analyses was filtered sequentially through polycarbonate filters with pore sizes of 10, 3, and 0.2 µm. The 0.2–3 µm size fraction was then used for sequencing and metaproteomics. Chlorophyll a concentrations measured via fluorescence and bacterioplankton cell counts measured via staining with 4′,6-diamidino-2-phenylindole were done as described previously [5, 6]. Phytoplankton abundance data are available via Pangaea at https://doi.pangaea.de/10.1594/PANGAEA.864676.

### Metagenome sequencing, assembly, and binning

Metagenome samples were sequenced at the Department of Energy Joint Genome Institute (JGI) (Walnut Creek, CA, USA) on the Illumina HiSeq 2500 platform using 2 × 150 bp chemistry. The automated metagenome assembly pipeline at the JGI using SPAdes v3.11.1 [43] for assembly and Prodigal v2.6.3 [44] and prokaryotic GeneMark.hmm version 2.8 [45] for gene prediction produced gene and amino acid predictions used for database construction for subsequent metaproteomics. These metagenome assemblies are available in the JGI GOLD database under the GOLD IDs Ga0206123–Ga0206131 (accessible at https://genome.jgi.doe.gov/portal/).

To maintain consistency with previous metagenomic and MAG generation work, metagenomes were also assembled and binned in house using the methods described in refs. [9, 10, 46], detail in Supplementary Text. MAGs were retained if at least one of either anvi’o [47] or CheckM [48] estimated completeness above 50% and contamination (CheckM) or redundancy (anvi’o) below 5%. Metagenome assemblies and MAGs from the four years 2010–2012 and 2016 are available in the European Nucleotide Archive under the project accession PRJEB28156.

### Metaproteome analyses—sample preparation

To extract total proteins from the 0.2 µm pore size filters, we used a modified protocol from ref. [49]. Detailed methods can be found in Supplementary Text, but in brief, filter pieces were shredded and transferred to low binding tubes (Sorenson BioScience, Salt Lake City, UT, USA) containing 100 µl resuspension buffer 1, then incubated in 150 µl resuspension buffer 2 for 10 min at 60 °C and 1200 rpm in a thermo-mixer (Eppendorf, Wesseling-Berzdorf, Germany). Next, 500 µl DNAse buffer was added and the cells lysed by ultrasonication and kept on ice. Lysate was incubated in the thermo-mixer for 10 min at 37 °C and 1200 rpm then centrifuged at 10,000 × *g* for 10 min at 4 °C.

Proteins were precipitated by adding pre-cooled trichloroacetic acid (TCA, 20% v/v). After centrifugation (12,000 × *g*, 30 min, 4 °C), the protein pellets were washed in pre-cooled (−20 °C) acetone (3 × 10 min, 12,000 × *g*, 4 °C) and dried by vacuum centrifugation at room temperature. Proteins were resuspended in 2× SDS sample buffer and separated by 1D PAGE, excised and trypsin-digested as described in ref. [6].

### Metaproteome analyses—liquid chromatography-tandem mass spectrometry (LC-MS/MS) analysis

Protein abundance was measured by LC-MS/MS using an Easy-nLC II (Thermo Fisher Scientific, Waltham, MA, USA) coupled to an LTQ Orbitrap Velos mass spectrometer (Thermo Fisher Scientific). For detailed methods, see Supplementary Text. In brief, samples were separated on capillary columns using a nonlinear binary gradient. The survey scan was carried out with a resolution of *R* = 60 k at *m*/*z* 400 followed by CID fragmentation of the 20 most abundant precursor ions (top20), and with dynamic exclusion enabled. Spectra were analysed using Mascot (Matrix Science, London, UK; version 2.6.0) to search the database of protein sequences predicted from the metagenome assemblies detailed above. Redundant sequences (99% identity) were removed using cd-hit [50], creating a database with 6,279,079 protein sequence entries, plus 42 common laboratory contaminants.

Scaffold v4.8.6 (Proteome Software Inc., Portland, OR, USA) was used to merge search results and validate MS/MS-based peptide and protein identifications. Peptide identifications were accepted if they could be established at greater than 95.0% probability by the Scaffold Local FDR algorithm. Protein identifications were accepted if they could be established at greater than 99.0% probability and contained at least two exclusive unique peptides. Protein probabilities were assigned by Protein Prophet [51]. Proteins that contained similar peptides and could not be differentiated based on MS/MS analysis alone were grouped.

For (semi-)quantitative analysis, percent normalised spectral abundance factor (%NSAF) values were calculated by dividing Scaffold’s ‘Quantitative Value’ for normalised, weighted (i.e. adjusted for protein size) spectra for each protein group, by the sum of all quantitative values for the sample as a whole [52]. Average values were calculated from three biological replicates (values for proteins that were not identified within a replicate were included as ‘0’ in the calculation).

Mass spectrometry proteomic data have been deposited with the ProteomeXchange Consortium via the PRIDE partner repository [53] and are available through the identifier PXD019294.

### Assignment of expressed proteins to MAGs

To assign identified expressed proteins a taxonomy, amino acid sequences were aligned using BLAST v2.5.0 [54] to all predicted proteins of all MAGs from both the corresponding metagenomes from 2016, and to the previously published MAGs from 2010 to 2012 [10]—also available in ENA project PRJEB28156. Alignments with identity >99% and *e*-value below E-4 were considered correctly assigned.

MAGs were clustered into approximate species groups using MASH v1.1.1 [55], classifying MAGs with MASH distance below 0.05 (functionally approximating average nucleotide identity above 95%) as belonging to the same species. Representative MAGs for each approximate species cluster were then chosen based on highest completeness, except when a substantially larger MAG (more than 100 kbp larger) and equivalent redundancy or contamination and near equivalent completeness was present in the cluster. In these cases, the larger MAG was chosen as representative. Protein sequences for MAGs were predicted using Prodigal v2.6.3 [44] within Prokka v1.12 [56]. Prokka was also used for automatic annotation of putative protein functions, and where possible, also to proteins where no matching MAG sequence could be found. Taxonomic identity of MAGs and thus predicted proteins was assigned using GTDB-Tk v0.3.1 [57] and v89 of the GTDB. GTDB-Tk incorporates pplacer v1.1 [58] for classification.

### Assignment of proteins to Pfam families and transporter classification database (TCDB) families

Assignment of Pfam annotations was done using the pfam_scan.pl pipeline and Pfam v33.1 [59]. Assignment to TCDB [60] families was done using BLAST against the TCDB, with *e*-value cutoff of E-10. For aggregate calculation of protein abundances, proteins were included in a category if they had a hit to either or both of Pfam or the TCDB.

### Prediction of TBDTs, *susD*-like genes, sulfatases, and CAZymes

In order to examine the polysaccharide transport-related function of detected proteins, we annotated TBDTs in the predicted proteins from the JGI metagenome assemblies used to create the metaproteome database. Details of the profiles used can be found in Supplementary Text, but briefly, sequences were compared to ten PFAM and TIGRFAM models using the hmmscan function of HMMER v3.2.1 [61] with default settings. Just one profile, TIGR04056, is the specific model for the SusC-like subclade of TBDTs. For PUL/CAZyme cluster function prediction, we used all MAGs included in PRJEB28156, in order to expand the potential for recovering well assembled PULs that could additionally be assigned to taxonomy. Sulfatases and SusD-like proteins were predicted with Prokka v1.12. CAZymes were assigned using the dbCAN v6 profiles [62]. HMM searches were performed using hmmscan as above for the TBDTs. Results were then filtered using the hmmscan-parser.sh script from dbCAN. CAZyme annotations were additionally confirmed using DIAMOND BLAST [63] searches of dbCAN-predicted CAZymes against the CAZy database v07312018 [64], in sensitive mode, with an *e*-value cutoff of E-20, minimum query cover of 40%, and minimum sequence identity of 30% [10]. This methodology is expected to cover known CAZyme families in the majority of clades; however, it cannot account for any biases in the representation of clades in the CAZy database.

### PUL prediction

PULs and other CAZyme-rich gene clusters were predicted by searching annotations of CAZymes and the surrounding upstream and downstream genes, using a seven-gene sliding window such that any CAZyme (excluding glycosyl transferases), sulfatase, TBDT, or *susD*-like genes would be grouped together if they were within seven genes of another such gene. We assume CAZyme annotations are not biased toward better predictions in individual taxonomic groups, based on the general diversity of taxa represented in the CAZy database. However, we cannot exclude the possibility that a systematic undersampling of CAZyme diversity exists for individual clades.

The subset of identified CAZyme containing gene clusters that contained a TBDT gene annotation was then taken forward for functional prediction. Functions themselves were predicted based on CAZyme content and known, characterised functions for the different CAZyme families as listed on the CAZy website (see Supplementary Text for specific CAZyme collections).

### TBDT classification based on phylogeny

We anticipate that there is functional conservation between closely related SusC-like proteins (e.g. [10, 15]) and assume this holds for TBDTs in general. We therefore reconstructed the phylogeny of our detected TBDTs and reference sequences identified in ref. [65]. We additionally included the protease secreting TBDT OAR in our reconstruction [66]. TBDT sequences were aligned with MAFFT L-INS-i v7.407 [67]. Spurious hits detected by the HMM searches but clearly not aligning with the majority collection of outer membrane TBDTs were removed and the 1261 remaining sequences were then realigned and their phylogeny reconstructed using FastTree v2.1 [68]. Trees were visualised with iTOL [69].

### Estimation of gene frequency of TBDTs and abundance of species representative MAGs

For estimation of gene and MAG abundance in the metagenomic datasets, SPAdes error corrected metagenomic reads were mapped to TBDT gene nucleotide sequences and species representative MAGs using BBMap v38.49 in ‘fast’ mode, with idfilter = 97 and minid = 99. Genes were taken from the 2016 JGI metagenome assemblies that were used to create the metaproteome database, except in cases where a longer and thus more complete equivalent was available in the MAG dataset. Read counts per gene were then converted to reads per kilobase per million (RPKM) values using the formula 1,000,000 × ((number of reads mapped ÷ length of gene/MAG in kilobase pairs) ÷ total number of reads in dataset).

### Monosaccharide composition of HMW-DOM

To assess the composition of the HMW-DOM over the course of the bloom, we collected 10 ml of the filtrate passed over the 10, 3 and 0.2 µm sequential filters, resulting in a total of 50 DOM samples. To remove salt and low-molecular weight DOM, 500 µl of sample or milliQ controls were dialysed in a Pur-A-Lyzer Midi Dialysis Kit with 1 kDa cutoff (PURD10005, Sigma Aldrich, St. Louis, MO, USA) according to manufacturer’s instructions. After dialysis, 500 µl of sample or control was hydrolysed with 500 µl of 2 M HCl at 100 °C for 24 h in pre-combusted (200 °C) glass ampoules (WHEA176772, VWR, Radnor, PA, USA). Next, two 200 µl aliquots of each acid hydrolysis were dried for 1 h at 30 °C in a vacuum concentrator (RVC 2-18 Cdplus, Martin Christ Gefriertrocknungsanlagen GmbH, Osterode am Harz, Germany) together with 100 µl aliquots of monosaccharide standard mix (Supplementary Table S1) prepared in 1 M HCl. Dried samples and standards were resuspended in 100 µl milliQ for subsequent high-performance anion-exchange chromatography with pulsed amperometric detection (HPAEC-PAD) analysis.

To quantify monosaccharides released by acid hydrolysis, we used a protocol for neutral and acidic sugars described previously [70]. In short, a Dionex ICS 5000+ ion chromatography system (Dionex Corp., Sunnyvale, CA, USA) with pulsed amperometric detection was equipped with a CarboPac PA10 analytical column (2 × 250 mm) and a CarboPac PA10 guard column (2 × 50 mm). Neutral and amino monosaccharides were separated by an isocratic flow of 18 mM NaOH for 20 min, followed by a gradient of 200 mM NaAcetate to separate acidic sugars. Standard substances listed in Supplementary Table S1 were used to identify peaks by retention time and a standard mix ranging from 1-10 to 1000 µg l^−1^ was used to quantify the amount of monosaccharide (*x*-axis amount and *y*-axis peak area).

## Results

### 2016 Helgoland phytoplankton bloom

**Fig. 1:**
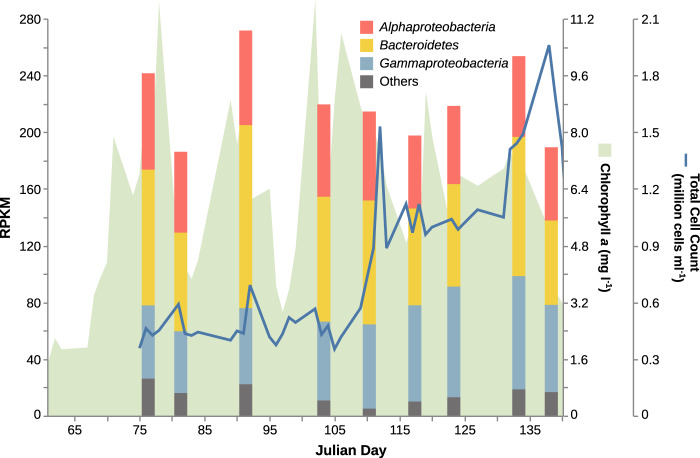
Abundance of representative MAGs during the 2016 bloom at Helgoland by taxonomic class. Abundances are reads per kilobase per million (RPKM). Chlorophyll a is taken as proxy for algal abundance. Total cell counts (TCC) are based on staining with 4′,6-diamidino-2-phenylindole.

The 2016 spring bloom at Helgoland was characterised by elevated chlorophyll a concentrations (between ~3 and ~10 µg l^−1^) throughout the sampling period (Fig. 1, data previously published in ref. [71]), implying a longer bloom than has been observed in prior years [5]. The composition of the algal community was similar to prior years [5], being dominated by diatoms (Supplementary Fig. S1). Total bacterioplankton cell counts (0.2–3 µm size fraction; approximately equivalent to ‘free living’ and non-eukaryotic rather than particle associated) increased rapidly between Julian days 105 and 112 from ~500,000 cells ml^−1^ in the early phase of the sampling period up to between one and two million cells ml^−1^ in the latter period (Fig. 1). This increase occurred shortly after the rise in diatom cell numbers (Supplementary Fig S1).

### Metagenomics and metaproteomics

The 2016 metagenomes were comparable to those from the years 2010–2012 [10], producing a set of MAGs with similar overall taxonomic composition, especially for the more abundant species. Clades of particular importance identified in prior studies were identified with similar numbers of species. These included SAR92 (MAGs from 15 species recovered in 2016 versus 14, 12, and 9 in 2010–2012, respectively), SAR86 (9 in 2016 versus 5, 12, and 12 in prior years), *Polaribacter* (8 in 2016; 6, 5, and 7 in prior years), and ‘Formosa’ clade Hel1-33-131 (6 in 2016; 9, 9, and 8 in prior years). Abundances based on read frequencies were also not unusual compared to earlier years (data not shown), consistent with the recurrence of bacterial clades previously reported for the region around Helgoland, where taxon abundance of *Bacteroidetes* and *Gammaproteobacteria* in particular has been observed to be similar from year to year [5, 8, 10]. The results of this study can thus be expected to be more generally applicable to spring blooms at Helgoland in other years.

From the six metaproteome samples, we identified 27,375 proteins (Supplementary Table S2). This represents 0.44% of the total predicted proteins from the metagenome assemblies. Of these, 17,806 (65.0%) could be assigned to a MAG and thus taxonomy. A total of 1261 (4.6%) of these proteins were identified as TBDTs (Supplementary Table S3). This compares with 3360 ribosomal proteins, 1324 ABC transporters, 877 ATP synthases, 600 RNA polymerases, 439 elongation factors, 376 TRAP transporters, 271 Hsp70 proteins, 267 porins, 257 chaperonins, and 228 SusD proteins.

### TBDT phylogeny and sequence diversity

Almost all of the detected TBDTs belonged to the *Bacteroidetes* and the *Gammaproteobacteria*—463 and 496 out of 1261, respectively, with 56 assigned to *Alphaproteobacteria*, four to *Verrucomicrobia*, one to *Actinobacteria*, and the remaining 241 not assigned to any MAG. BLAST results against the NCBI nr database (as of October 15, 2019) classified 116 of these 241 as *Gammaproteobacteria*, 83 as *Bacteroidetes*, 16 as *Alphaproteobacteria*, and one as *Verrucomicrobia*, with the remaining 25 not successfully aligned. The total for *Bacteroidetes* is slightly underestimated as three of the SusC-like proteins (*Bacteroidetes* specific) were binned incorrectly into alphaproteobacterial MAGs of the order *Pelagibacterales*—likely the result of coincidental overlap in abundance profiles.

**Fig. 2:**
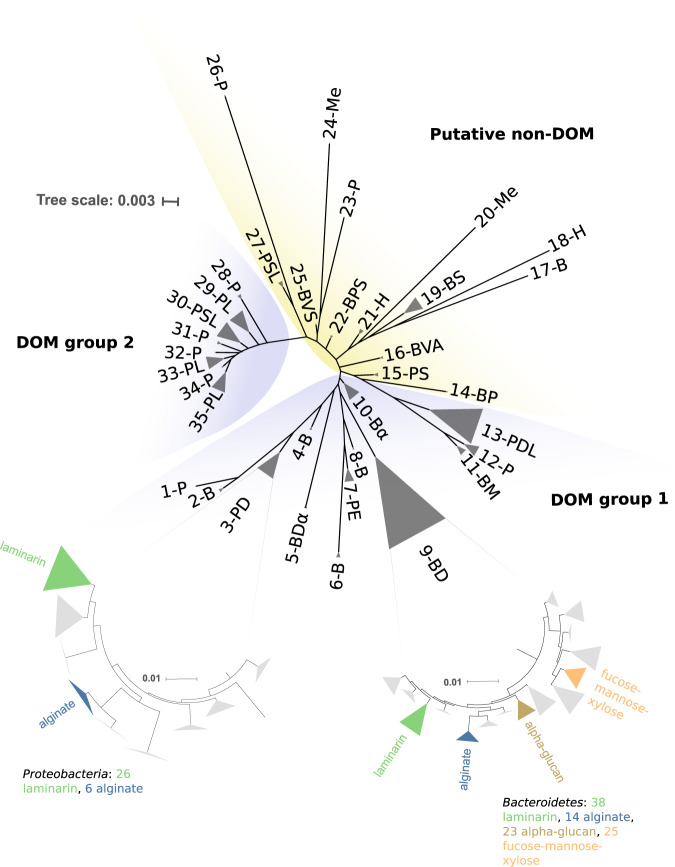
Phylogeny of 1261 detected expressed TonB-dependent transporters in the metaproteome data, the 96 reference TBDT sequences with known substrates from ref. [65], and the peptidase exporter from ref. [66], divided into the four categories ascribed therein, namely DOM transporters, metals transporters, haemophore transporters, and siderophore transporters. The three lobes (two blue, one yellow) are putative functional groupings. DOM group 1 includes 844 sequences, 64 associated with laminarin degrading CAZymes, 20 with alginate, 23 with alpha-glucan, 25 with fucose, mannose, or xylose-containing polymers, 53 associated with CAZymes where no substrate was predicted, 22 reference DOM transporters, and the peptidase transporter reference. DOM group 2 includes 338 proteobacterial sequences, 7 associated with laminarin degrading CAZymes, 17 with other CAZymes without substrate predicted, and 4 reference siderophore transporters. The putative non-DOM clade includes 182 sequences, none associated with CAZymes. Of those 182, 69 are reference sequences, all of them siderophore, haem, or metals transporters. Individual clades in the tree are arbitrarily labelled numerically for findability, and with letter codes indicating the types of sequences in that clade. B = sequences from *Bacteroidetes*, P = sequences from *Proteobacteria*, A = sequences from *Actinobacteria*, V = sequences from *Verrucomicrobia*; D = DOM transporter reference, E = exporter reference, H = haem transporter reference, Me = metals transporter reference, S = siderophore transporter reference; α = alpha-glucan transporter, L = laminarin transporter, M = mannose-rich polymer transporter. All clades can be associated with sequences in the data in Supplementary Table S3. Clades 3-PD and 9-BD correspond to the main PUL-containing clades of TBDTs, and thus the polysaccharide transporters are left out of the label encoding in favour of the expanded subtrees with additional detail. Clade 9-BD corresponds to the SusC-like proteins, identified by TIGR04056.

The phylogenetic reconstruction of TBDTs (Fig. 2) broadly divides into three lobes (not perfectly monophyletic), which we henceforth refer to as DOM groups 1 and 2 and putative non-DOM. While molecules such as siderophore are technically DOM, we use the term DOM transporter to refer specifically to DOM transported as an energy source, and non-DOM for transport for other purposes. The putative non-DOM transporters include all but four of the reference sequences from ref. [65] for transporters of siderophores, vitamins, metals, and haemophores, along with three CAZyme-associated sequences, one with predicted laminarin transport function and the others without specific predicted polysaccharide substrate (in the clades 27-PSL, 22-BPS, and 25-BVS, respectively, Fig. 2). The DOM groups include all reference sequences for transporters of organic molecules used as energy sources, while DOM group 2 also includes four reference siderophore transporters (clade 30-PSL, Fig. 2).

DOM transporters make up the vast majority of the detected proteins in our dataset, the single largest subset of which is the SusC-like clade (clade 9-BD, Fig. 2), with 306 sequences. For the majority of SusC-like proteins detected in the metaproteomes (216 of 306), an adjacent SusD-like partner was also annotated. Of the remaining 90, 62 were not assigned to a MAG and thus the presence or absence of a SusD-like partner was not determined. Examples where the SusD-like partner is present extend throughout the SusC-like clade, thus we might expect that almost all SusC-like proteins should have an associated SusD-like protein. Within the SusC-like clade, 67 of the detected transporters have a clearly associated and determinable polysaccharide substrate or class of substrates, while a further 18 have at least some CAZyme association (Table 1).

**Table 1:** Expression of TBDTs (divided by whether they are part of the SusC-like subclade (9-BD in Fig. 2) or not) in different categories and their representation among all TBDT sequences.

Category	Number of sequences	Percent of all TBDT sequences	Average NSAF%	Percent of TBDT NSAF%
SusC-like with polysaccharide substrate predicted	67	5.3	1.6	9.8
SusC-like with CAZyme but no substrate predicted	18	1.4	0.6	3.7
SusC-like with no CAZymes	221	17.5	3.8	22.7
Not SusC-like with polysaccharide substrate predicted	28	2.2	0.4	2.3
Not SusC-like with CAZyme but no substrate predicted	54	4.3	0.8	5.0
Not SusC-like with no CAZymes	873	69.2	9.4	56.5

TBDT sequences constitute an average 16.7% of total NSAF across the six samples.

Substrate-specific clustering of *Bacteroidetes* SusC-like sequences has been demonstrated previously [10, 15, 72]. Based on this, one may infer polysaccharide substrates based on sequence similarity for those sequences where CAZymes were not found in the genomic neighbourhood, but where there was a clearly defined and close phylogenetic grouping (clade 9-BD, Fig. 2). This concept also applies partially to gammaproteobacterial TBDTs (clade 3-PD, Fig. 2). One important caveat here is that a number of apparently laminarin-associated TBDTs in the *Gammaproteobacteria* fall outside the primary laminarin-associated clade in DOM group 1. But there are defined clades for proteobacterial alginate and laminarin PULs (expanded subtree for clade 3-PD, Fig. 2) that contain all proteobacterial sequences that are alginate associated and ten of the laminarin-associated sequences. Nine more distantly related proteobacterial predicted laminarin transporters are found in other parts of DOM groups 1 and 2 (clades encoded with the letters P and L, Fig. 2).

One final clade of note among the putative DOM transporters is the gammaproteobacterial clade 7-PE in Fig. 2, containing the recently described reference peptidase exporting TBDT of a *Myxococcus* species [66]. This is a smaller clade (45 sequences), where within-clade sequence identities are low (between 30 and 40%, and below 30% between the reference and all others in the clade). However, the presence of the reference sequence is a reminder of the possibility of other functions for TBDTs, although with low sequence identities here we cannot in good faith infer such a function.

### TBDT protein abundance

**Fig. 3:**
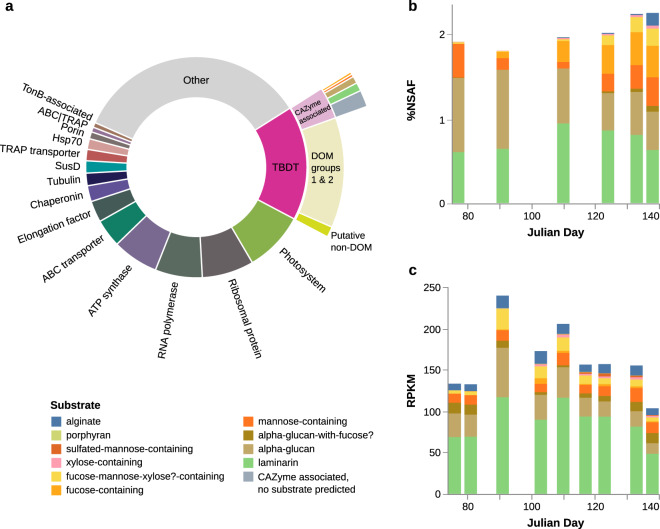
Abundance of TBDTs in metaproteomic and metagenomic datasets by substrate targeted. **a** Average abundance of the most abundant categories of proteins across the six sampling dates, with TonB-dependent transporters (TBDT) subdivided further into average abundance of polysaccharide-transporting proteins (outermost ring indicates individual substrate categories where large enough to be included). **b** TBDTs where substrate could be assigned, based on proximity of CAZymes in MAGs. **c** Gene frequency (reads per kilobase per million—RPKM) of TBDTs where substrate could be assigned using CAZyme proximity. Note omission of CAZyme associated, no substrate predicted proteins from **b** and **c**, and that values in **c** are not directly comparable with those in Fig. 1, as they are cumulative over individual genes, not entire genomes.

Between 17 March and 17 May 2016, combined abundance of all TBDTs (as a proportion of the total quantified protein, regardless of whether attributed to a MAG) went from 13.9% normalised spectral abundance factor (%NSAF) to its maximum in the final sample of 21.0 %NSAF, with expression reasonably equally split between the *Bacteroidetes* and the *Gammaproteobacteria* (Supplementary Fig. S2a). Across all six samples, TBDTs accounted for an average 16.7% of all detected proteins (s.d. = 3.94%) (Fig. 3a). This is the highest expressed single category of proteins. For comparison, the next most abundant categories were ribosomal proteins, making up an average 7.6% of all protein (s.d. = 0.78%), RNA polymerases making up 6.9% (s.d. = 1.06%), and ATP synthases making up 6.7% (s.d. = 1.99%) (Fig. 3a, Supplementary Fig. S3). The most abundant classes of transporters other than TBDTs were the ABC transporters, TRAP transporters, and porins, which had average %NSAF values of 4.1% (s.d. = 0.65%), 1.7% (s.d. = 0.41%), and 1.0% (s.d. = 0.53%), respectively. In contrast to the overall TBDT abundance, TBDTs which might play a role in siderophore, vitamin, or metal uptake had low abundances (average 1.15% NSAF, s.d. = 0.16%) and did not trend up or down (Figs. 2 and  3a, Supplementary Fig. S2b).

While overall abundance of TBDTs was evenly divided between the *Bacteroidetes* and the *Gammaproteobacteria*, among those with a specific predicted polysaccharide substrate or substrate class, the cumulative %NSAF for *Gammaproteobacteria* was between approximately one-fifth and one-third as large as that for *Bacteroidetes*. Together these TBDTs had average %NSAF of 2.02 (s.d. = 0.18%) (Fig. 3a, b). Average abundance of TBDTs associated with CAZymes without a clearly predictable substrate was 1.45% (s.d. = 0.33%, Fig. 3a, b), see Supplementary Text for further details.

### Polysaccharide-associated TBDT gene frequency and expression

When looking in detail at transporters where a polysaccharide substrate could be predicted, we detected seemingly non-random changes in protein abundance over time (Fig. 3b), although it should be noted that the still small number of biological samples, which precludes statistical analysis of effect size, leaves the true magnitude and interannual reproducibility of the trend uncertain. Early in the bloom, laminarin and alpha-glucan associated TBDTs were the most abundant polysaccharide transporters, while as the bloom progressed we saw increases in the %NSAF of transporters associated with fucose-, mannose-, and xylose-containing polymers (initially dropping from 0.4% of proteins to 0.2%, then increasing to 1.0% of all proteins by the final time point). Furthermore, we see an approximately tenfold increase in the %NSAF of predicted alginate transporters on the final sampling date (from 0.01 to 0.1% of all proteins). The increase in the %NSAF of TBDTs predicted to transport mannose-containing polysaccharides followed an initial dip from when transporters were more abundant early in the bloom (Fig. 3b). This specifically mannose-polymer-associated TBDT expression was almost entirely caused by a single abundant species within the NS4 clade (GTDB: MAG-121220-bin8, Supplementary Table S2). What is most striking, however, is that while the patterns for laminarin and alpha-glucans appear broadly similar in the gene frequency analysis (Fig. 3c), they are quite different for the fucose-, mannose-, and xylose-containing polysaccharides. Here, the frequency of the genes encoding these proteins did not change strongly over time. Genes encoding alginate transporters were present at a similar frequency throughout the bloom (average RPKM = 11.2), as were those for mannose-polymer transporters (average RPKM = 11.7). Combined fucose-, mannose-, and xylose-containing polysaccharide transporters even appeared to decline in significance relative to other TBDTs when looking at gene frequencies (max. RPKM = 33.9 on day 110, declines to 16.6 by day 138), which is the opposite of what was apparent from the metaproteome data.

**Fig. 4:**
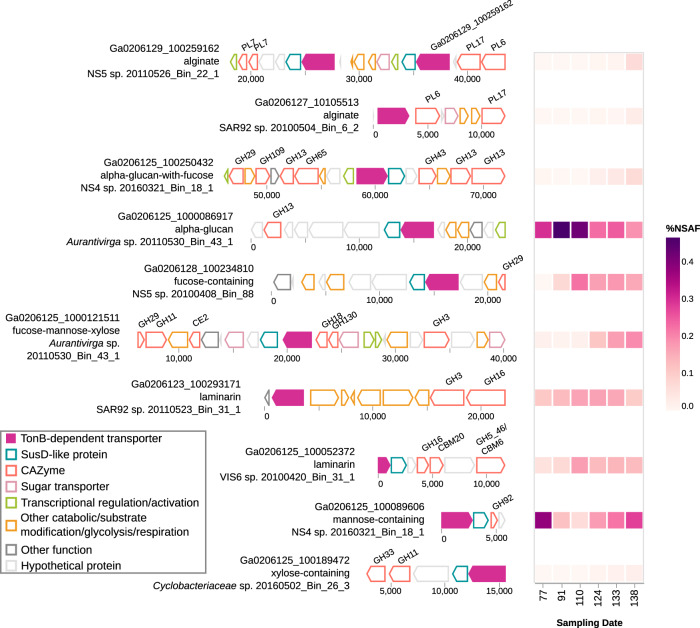
PUL structures and protein abundance of the most abundant TonB-dependent transporters in metaproteomic samples, for each polysaccharide substrate class where substrate could be assigned. PUL gene arrangements were taken from individual MAGs where they were clearest, which in most cases were not the species representative MAG. The top-most TBDT, Ga0206129_100259162, is part of a ‘tandem-PUL’ structure, with two *susCD*-like genes between the two pairs of alginate specific polysaccharide lyases.

This pattern of late onset expression of non-glucose polymer targeting transporters was also clear when looking at the individual PULs with the highest expression in each category (Fig. 4). Protein abundance increased over time for the predicted alginate, alpha-glucan with fucose, fucose/mannose/xylose-containing, and xylose-containing polysaccharide transporters. This indicates that this is not only an aggregate effect of the whole community, but also an active change made by individual species or populations. This is perhaps best demonstrated by *Aurantivirga* sp. 20110530_Bin_43_1, which early on in the bloom had the most abundant putative alpha-glucan transporter (Fig. 4). The abundance of this putative alpha-glucan transporter fell in %NSAF terms over time however, and concurrently the fucose/mannose/xylose polymer-transporting SusC-like protein in this species became more prominent (Fig. 4).

### Species-specific expression

Observing individual species and their gene expression, multiple TBDTs belonging to single species of both *Bacteroidetes* and *Gammaproteobacteria* (maximum 41, mean = 6.46 per species, s.d. = 6.34) were detected. Only rarely was the most highly abundant TBDT for an individual species one with an assigned polysaccharide substrate (Supplementary Fig. S3). The most abundant single proteins were SusC-like proteins belonging to species of the *Bacteroidetes* genus *Aurantivirga*, while the largest numbers of detected proteins from individual genomes were in species of the gammaproteobacterial genus *Luminiphilus*. For further detail on individual species see also Supplementary Text and Supplementary Fig. S4.

### Monosaccharide composition of HMW-DOM

**Fig. 5:**
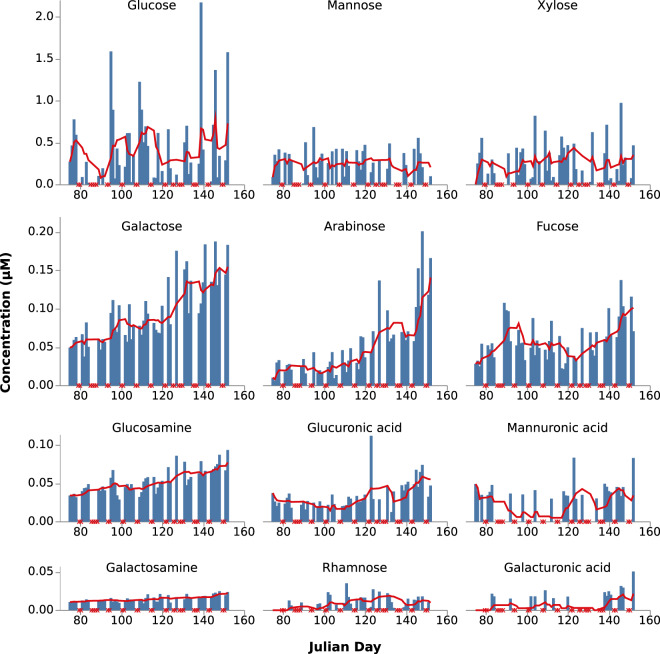
Concentrations of monosaccharide components of high molecular weight dissolved organic matter measured by acid hydrolysis and high-performance anion-exchange chromatography with pulsed amperometric detection over the course of the 2016 spring bloom. Bars are raw values. Dates where samples were not collected and thus measurements not made are indicated on the *x*-axis with an ‘X’. Red lines are five-sample trailing average values which exclude dates where samples were not collected.

The measurement of monosaccharide composition of HMW-DOM determined via acid hydrolysis showed the most abundant monomers to be glucose, xylose, and mannose (Fig. 5, Supplementary Table S4). These three monomers were approximately one order of magnitude higher in concentration (maxima—2.2, 1.0, and 0.7 µM, respectively) compared to the sum of other monomers measured (maximum 0.11 µM for all others combined). Excluding glucose, xylose, and mannose, the five-sample trailing averages indicate a general accumulation of most of the lower abundant sugars over the sampling period, with an approximately threefold increase in concentration from the early period to Julian day 110 to the peak at the end of the sampling period at Julian day 152. As was the case for the metaproteome sampling, the limitations in generating replicates preclude a more robust statistical analysis of the effect size of the trends observed. Nevertheless, this increase directly coincides temporally with the increase in bacterial cell counts (Fig. 1). Abundance of glucose, xylose, and mannose were more variable and did not clearly increase or decrease over the course of the bloom.

## Discussion

TBDTs predicted to be specific for polysaccharide uptake are known to be abundant during spring blooms [10, 15]; however, to date, there has been insufficient temporal resolution in metaproteome data to identify trends in protein abundance. Here, we identified an apparent trend in our data that indicated a shift in abundance of TBDTs with predicted polysaccharide substrates, in favour of transport of more complex molecules during the later stages of the bloom. This provides an important indication, at least from this one year, in favour of the hypothesis that bacterial polysaccharide consumption changes as part of an ecological succession predicated on the availability of different polysaccharide niches.

TBDTs were the single most abundant protein family observed during the 2016 spring bloom at Helgoland, and accounted for a maximum of 21% of detected expressed protein. This proportion is consistent with previous reports (e.g. [6, 41]). Only a small part of the TBDT abundance was associated with putative ion or vitamin acquisition functions (average 1.2%), with the great majority thus putatively associated with uptake of DOM. This reinforces the notion that uptake via outer membrane transporters is the dominant means of accessing algal organic matter and thus energy for heterotrophic members of the bacterial spring bloom community, and that the diderm cell organisation with transporters situated in the outer membrane represents a key innovation of Gram-negative bacteria such as *Bacteroidetes* and *Gammaproteobacteria*. This remarkable proportion of proteins dedicated to uptake illustrates how much DOM is available to be exploited by the bacterioplankton community during algal blooms. The increase in the proportion of total %NSAF dedicated to TBDTs over the course of the bloom, which, as measurement of monosaccharide components of HMW-DOM suggests, coincides with greater availability of organic matter as more algae die, further reiterates this point.

Between 23 and 28% of total TBDT abundance, or an average of 3.5% of all detected expressed protein, was linked to transporters with putative glycan transporting functions. This highlights both the significance of polysaccharide as an energy source for members of the *Bacteroidetes* and *Gammaproteobacteria*, and also that there are still considerable unknowns regarding the function of the otherwise unclassified TBDTs that potentially take up other components of DOM.

The abundance of putatively polysaccharide-transporting TBDTs was skewed toward the *Bacteroidetes*, and their characteristic SusC-like proteins. To date, members of this protein family have only been demonstrated to transport oligosaccharides, with putative peptide and nucleotide transport functions also having been suggested [18, 33]. There are several possible explanations for the disparity in abundances of identified putative polysaccharide transporters between the *Bacteroidetes* and *Gammaproteobacteria*. The most basic is that the *Bacteroidetes* are more likely to consume polysaccharide, with *Gammaproteobacteria* more focused on other parts of DOM. Alternatively, it could be the case that the clustering of CAZymes close to the relevant transporter is less common in gammaproteobacterial genomes, or, perhaps less likely, a simple bias in annotation of CAZymes leaves us poorly equipped to identify polysaccharide consumption capacity to the same extent as in *Bacteroidetes*. Assuming no bias in our identification of putative polysaccharide transporters, however, without direct characterisation of transporter specificities, monitoring of these uptake processes via transporter protein abundance can only be approximate.

The shift toward transporters for putatively harder to degrade algal cell wall-associated fucose-, mannose-, and xylose-containing polysaccharides and also alginates did not show an obvious relationship with changes in the abundance of the corresponding xylose, mannose, and mannuronic acid monomers measured. However, this change did coincide with the observed increase in abundance of the rare monosaccharides in HMW-DOM (e.g. arabinose, galactose, fucose, and glucosamine). This may be indicative of a rise in the overall complexity of the polysaccharides present, which at the very least complements the observed change in polysaccharide transporter abundance, despite the fact that the predicted transported substrates are not polymers of these individual saccharides. Replication of this pattern and a better understanding of the polysaccharides present in DOM following algal blooms would thus be required in order to confirm any direct relationship between transporter abundance and the presence of different polysaccharides. However, this abundance pattern is at least consistent with the temporal shifts in polysaccharide concentration previously observed in particulate organic matter during a spring bloom [73]. The shift in transporter expression is of course in relative abundance, and does not necessarily indicate an absolute change in the expression of transporters for storage polysaccharides in this period. Instead the fall in proportion of total %NSAF dedicated to laminarin and alpha-glucans may simply reflect the clear upward trend in abundance of both transporters for other polysaccharide substrates and for putative transporters of DOM without a predicted substrate. At the very least, the measurement of monosaccharide compositions confirms the presence of HMW material of a composition consistent with that predicted from the genomic and expression data, even if the trends observed are less easily interpreted.

The change in monosaccharide composition during the bloom could also potentially have been caused by a change in phytoplankton community composition. For example, the sudden drop and subsequent rise of diatom cell numbers in the latter half of the bloom might have been caused by a loss of one taxon followed by growth of another. If a change in algal species did occur and with it a change of polysaccharides presented to the bacteria, this could also explain the observed change in polysaccharide specificities of TBDTs. Demonstrating this would, however, require a deeper understanding of the polysaccharides produced by different algal species under the conditions present during blooms, which to date is lacking.

There are also a number of other possible scenarios that would be consistent with the observed trends in polysaccharide transporter abundance. One option is that accumulation of dissolved cell wall-derived polysaccharide over time directly induced the increase in expression of transporters for these molecules (i.e. their consumption is only worthwhile once they reach a certain concentration). Alternatively, co-induction could have occurred as a result of general increases in DOM concentrations, meanwhile greater bacterial cell numbers might have resulted in a reduction in DOM concentration per bacterial cell, enhancing competition and favouring consumption of less easily degradable material.

Recent in vitro studies have also found that the marine gammaproteobacterium *Alteromonas macleodii* 83-1, when provided with a mix of laminarin, alginate, and pectin, prioritises laminarin before switching to simultaneous alginate and pectin utilisation once laminarin has been exhausted [74]. This biphasic phenotype went hand in hand with pronounced shifts in specific PUL gene expression patterns. This phenomenon resembles catabolite repression mechanisms of terrestrial bacteria consuming plant-derived sugars [75]. Something resembling a prioritisation of substrates might therefore explain the observed shifts in transporter abundances during the spring bloom.

Finally, we cannot discount unknown other factors favouring particular bacterial clades that coincidentally have differing polysaccharide preferences. Our observations cover only a single spring bloom, and thus it would be very interesting to see if the patterns we see can be generalised to other phytoplankton blooms. The data are, at the very least, consistent with the observations made with lower sampling densities from previous years at Helgoland [10, 15].

The observed trends in protein abundance detected during the 2016 spring bloom were not reflected in the corresponding metagenomic data. Genes for transporters of alginate, for example, were present in similar frequency throughout the bloom, despite clear expression only being realised in the final metaproteomic sample. This confirms that metagenomic data alone are insufficient to track polysaccharide utilisation during spring phytoplankton blooms, and expression and protein abundance data can be a better proxy for tracking bacterial responses to phytoplankton blooms.

Thus, the quantification of transporter proteins could indeed become an important piece in solving the highly complex puzzle of marine carbon cycling. However, to further elucidate the ecological processes at play here would require first of all a biochemical validation of the substrates which are taken up by the various TBDTs. Enzymatic quantification of distinct polysaccharides (e.g. laminarin [76, 77]) and not just of linkage types and monomers would be another requirement. Ultimately, it will be the combination of various methods that will advance our knowledge of the molecules, enzymatic reactions, and rates underlying the marine carbon cycle, which is a prerequisite for predicting and managing atmospheric carbon dioxide levels.

## Supplementary information

Supplementary Figure S1

Supplementary Figure S2

Supplementary Figure S3

Supplementary Figure S4

Supplementary Table S1

Supplementary Table S2

Supplementary Table S3

Supplementary Table S4

Supplementary text

## References

[1] Behrenfeld MJ, Randerson JT, McClain CR, Feldman GC, Los SO, Tucker CJ (2001). Biospheric primary production during an ENSO transition. Science..

[2] Buchan A, LeCleir GR, Gulvik CA, González JM (2014). Master recyclers: features and functions of bacteria associated with phytoplankton blooms. Nat Rev Microbiol.

[3] Field CB, Behrenfeld MJ, Randerson JT, Falkowski P (1998). Primary production of the biosphere: integrating terrestrial and oceanic components. Science..

[4] Needham DM, Fuhrman JA (2016). Pronounced daily succession of phytoplankton, archaea and bacteria following a spring bloom. Nat Microbiol.

[5] Teeling H, Fuchs BM, Bennke CM, Krüger K, Chafee M, Kappelmann L (2016). Recurring patterns in bacterioplankton dynamics during coastal spring algae blooms. eLife..

[6] Teeling H, Fuchs BM, Becher D, Klockow C, Gardebrecht A, Bennke CM (2012). Substrate-controlled succession of marine bacterioplankton populations induced by a phytoplankton bloom. Science..

[7] Williams TJ, Wilkins D, Long E, Evans F, DeMaere MZ, Raftery MJ (2013). The role of planktonic *Flavobacteria* in processing algal organic matter in coastal East Antarctica revealed using metagenomics and metaproteomics. Environ Microbiol.

[8] Chafee M, Fernàndez-Guerra A, Buttigieg PL, Gerdts G, Eren AM, Teeling H (2018). Recurrent patterns of microdiversity in a temperate coastal marine environment. ISME J.

[9] Francis TB, Krüger K, Fuchs BM, Teeling H, Amann RI (2019). Amann RI. *Candidatus*Prosiliicoccus vernus, a spring phytoplankton bloom associated member of the *Flavobacteriaceae*. Syst Appl Microbiol.

[10] Krüger K, Chafee M, Francis TB, Glavina del Rio T, Becher D, Schweder T (2019). In marine *Bacteroidetes* the bulk of glycan degradation during algae blooms is mediated by few clades using a restricted set of genes. ISME J.

[11] Needham DM, Fichot EB, Wang E, Berdjeb L, Cram JA, Fichot CG (2018). Dynamics and interactions of highly resolved marine plankton via automated high-frequency sampling. ISME J.

[12] Cottrell MT, Kirchman DL (2000). Natural assemblages of marine Proteobacteria and members of the *Cytophaga*-*Flavobacter* cluster consuming low- and high-molecular-weight dissolved organic matter. Appl Environ Microbiol.

[13] Fernández-Gomez B, Richter M, Schüler M, Pinhassi J, Acinas SG, González JM (2013). Ecology of marine *Bacteroidetes*: a comparative genomics approach. ISME J..

[14] Grondin JM, Tamura K, Déjean G, Abbott DW, Brumer H (2017). Polysaccharide utilization loci: fueling microbial communities. J Bacteriol.

[15] Kappelmann L, Krüger K, Hehemann J-H, Harder J, Markert S, Unfried F (2019). Polysaccharide utilization loci of North Sea Flavobacteriia as basis for using SusC/D-protein expression for predicting major phytoplankton glycans. ISME J.

[16] Kirchman DL (2002). The ecology of *Cytophaga*–*Flavobacteria* in aquatic environments. FEMS Microbiol Ecol.

[17] Thomas F, Hehemann J-H, Rebuffet E, Czjzek M, Michel G (2011). Environmental and gut *Bacteroidetes*: the food connection. Front Microbiol.

[18] Glenwright AJ, Pothula KR, Bhamidimarri SP, Chorev DS, Baslé A, Firbank SJ (2017). Structural basis for nutrient acquisition by dominant members of the human gut microbiota. Nature..

[19] Joglekar P, Sonnenburg ED, Higginbottom SK, Earle KA, Morland C, Shapiro-Ward S (2018). Genetic variation of the SusC/SusD homologs from a polysaccharide utilization locus underlies divergent fructan specificities and functional adaptation in *Bacteroides thetaiotaomicron* strains. mSphere..

[20] Cuskin F, Lowe EC, Temple MJ, Zhu Y, Cameron EA, Pudlo NA (2015). Human gut *Bacteroidetes* can utilize yeast mannan through a selfish mechanism. Nature..

[21] Reintjes G, Arnosti C, Fuchs BM, Amann R (2017). An alternative polysaccharide uptake mechanism of marine bacteria. ISME J.

[22] Hehemann J-H, Truong LV, Unfried F, Welsch N, Kabisch J, Heiden SE (2017). Aquatic adaptation of a laterally acquired pectin degradation pathway in marine Gammaproteobacteria. Environ Microbiol.

[23] Neumann AM, Balmonte JP, Berger M, Giebel H-A, Arnosti C, Voget S (2015). Different utilization of alginate and other algal polysaccharides by marine *Alteromonas macleodii* ecotypes. Environ Microbiol.

[24] Mirus O, Strauss S, Nicolaisen K, von Haeseler A, Schleiff E (2009). TonB-dependent transporters and their occurrence in Cyanobacteria. BMC Biol.

[25] Gudmundsdottir A, Bell PE, Lundrigan MD, Bradbeer C, Kadner RJ (1989). Point mutations in a conserved region (TonB box) of *Escherichia coli* outer membrane protein BtuB affect vitamin B12 transport. J Bacteriol.

[26] Köster W, Braun V (1990). Iron (III) hydroxamate transport into *Escherichia coli*. Substrate binding to the periplasmic FhuD protein. J Biol Chem.

[27] Schauer K, Gouget B, Carrière M, Labigne A, Reuse HD (2007). Novel nickel transport mechanism across the bacterial outer membrane energized by the TonB/ExbB/ExbD machinery. Mol Microbiol.

[28] Reeves AR, D’Elia JN, Frias J, Salyers AA (1996). A Bacteroides thetaiotaomicron outer membrane protein that is essential for utilization of maltooligosaccharides and starch. J Bacteriol.

[29] Cheng Q, Yu MC, Reeves AR, Salyers AA (1995). Identification and characterization of a Bacteroides gene, csuF, which encodes an outer membrane protein that is essential for growth on chondroitin sulfate. J Bacteriol.

[30] Neugebauer H, Herrmann C, Kammer W, Schwarz G, Nordheim A, Braun V (2005). ExbBD-dependent transport of maltodextrins through the novel MalA protein across the outer membrane of *Caulobacter crescentus*. J Bacteriol.

[31] Noinaj N, Guillier M, Barnard TJ, Buchanan SK (2010). TonB-dependent transporters: regulation, structure, and function. Annu Rev Microbiol.

[32] Schauer K, Rodionov DA, de Reuse H (2008). New substrates for TonB-dependent transport: do we only see the ‘tip of the iceberg’?. Trends Biochem Sci.

[33] Lapébie P, Lombard V, Drula E, Terrapon N, Henrissat B (2019). *Bacteroidetes* use thousands of enzyme combinations to break down glycans. Nat Commun.

[34] Cantarel BL, Coutinho PM, Rancurel C, Bernard T, Lombard V, Henrissat B (2009). The Carbohydrate-Active EnZymes database (CAZy): an expert resource for Glycogenomics. Nucleic Acids Res.

[35] Foley MH, Cockburn DW, Koropatkin NM (2016). The Sus operon: a model system for starch uptake by the human gut *Bacteroidetes*. Cell Mol Life Sci.

[36] Terrapon N, Lombard V, Gilbert HJ, Henrissat B (2015). Automatic prediction of polysaccharide utilization loci in *Bacteroidetes* species. Bioinformatics..

[37] Terrapon N, Lombard V, Drula E, Lapébie P, Al-Masaudi S, Gilbert HJ (2017). PULDB: the expanded database of polysaccharide utilization loci. Nucleic Acids Res.

[38] Bergauer K, Fernandez-Guerra A, Garcia JA, Sprenger RR, Stepanauskas R, Pachiadaki MG (2018). Organic matter processing by microbial communities throughout the Atlantic water column as revealed by metaproteomics. Proc Natl Acad Sci USA.

[39] Dong H-P, Hong Y-G, Lu S, Xie L-Y (2014). Metaproteomics reveals the major microbial players and their biogeochemical functions in a productive coastal system in the northern South China Sea. Environ Microbiol Rep.

[40] McCarren J, Becker JW, Repeta DJ, Shi Y, Young CR, Malmstrom RR (2010). Microbial community transcriptomes reveal microbes and metabolic pathways associated with dissolved organic matter turnover in the sea. Proc Natl Acad Sci USA.

[41] Morris RM, Nunn BL, Frazar C, Goodlett DR, Ting YS, Rocap G (2010). Comparative metaproteomics reveals ocean-scale shifts in microbial nutrient utilization and energy transduction. ISME J.

[42] Williams TJ, Long E, Evans F, DeMaere MZ, Lauro FM, Raftery MJ (2012). A metaproteomic assessment of winter and summer bacterioplankton from Antarctic Peninsula coastal surface waters. ISME J.

[43] Nurk S, Meleshko D, Korobeynikov A, Pevzner PA (2017). metaSPAdes: a new versatile metagenomic assembler. Genome Res.

[44] Hyatt D, Chen G-L, LoCascio PF, Land ML, Larimer FW, Hauser LJ (2010). Prodigal: prokaryotic gene recognition and translation initiation site identification. BMC Bioinform.

[45] Besemer J, Lomsadze A, Borodovsky M (2001). GeneMarkS: a self-training method for prediction of gene starts in microbial genomes. Implications for finding sequence motifs in regulatory regions. Nucleic Acids Res.

[46] Orellana LH, Francis TB, Krüger K, Teeling H, Müller M-C, Fuchs BM (2019). Niche differentiation among annually recurrent coastal Marine Group II Euryarchaeota. ISME J.

[47] Eren AM, Esen ÖC, Quince C, Vineis JH, Morrison HG, Sogin ML (2015). Anvi’o: an advanced analysis and visualization platform for ‘omics data. PeerJ..

[48] Parks DH, Imelfort M, Skennerton CT, Hugenholtz P, Tyson GW (2015). CheckM: assessing the quality of microbial genomes recovered from isolates, single cells, and metagenomes. Genome Res.

[49] Deusch S, Seifert J (2015). Catching the tip of the iceberg—evaluation of sample preparation protocols for metaproteomic studies of the rumen microbiota. Proteomics..

[50] Li W, Godzik A (2006). Cd-hit: a fast program for clustering and comparing large sets of protein or nucleotide sequences. Bioinformatics..

[51] Nesvizhskii AI, Keller A, Kolker E, Aebersold R (2003). A statistical model for identifying proteins by tandem mass spectrometry. Anal Chem.

[52] Florens L, Carozza MJ, Swanson SK, Fournier M, Coleman MK, Workman JL (2006). Analyzing chromatin remodeling complexes using shotgun proteomics and normalized spectral abundance factors. Methods..

[53] Perez-Riverol Y, Csordas A, Bai J, Bernal-Llinares M, Hewapathirana S, Kundu DJ (2019). The PRIDE database and related tools and resources in 2019: improving support for quantification data. Nucleic Acids Res.

[54] Altschul SF, Madden TL, Schäffer AA, Zhang J, Zhang Z, Miller W (1997). Gapped BLAST and PSI-BLAST: a new generation of protein database search programs. Nucleic Acids Res.

[55] Ondov BD, Treangen TJ, Melsted P, Mallonee AB, Bergman NH, Koren S (2016). Mash: fast genome and metagenome distance estimation using MinHash. Genome Biol.

[56] Seemann T (2014). Prokka: rapid prokaryotic genome annotation. Bioinformatics..

[57] Parks DH, Chuvochina M, Waite DW, Rinke C, Skarshewski A, Chaumeil P-A (2018). A standardized bacterial taxonomy based on genome phylogeny substantially revises the tree of life. Nat Biotechnol.

[58] Matsen FA, Kodner RB, Armbrust EV (2010). pplacer: linear time maximum-likelihood and Bayesian phylogenetic placement of sequences onto a fixed reference tree. BMC Bioinform.

[59] El-Gebali S, Mistry J, Bateman A, Eddy SR, Luciani A, Potter SC (2019). The Pfam protein families database in 2019. Nucleic Acids Res.

[60] Saier MH, Reddy VS, Tsu BV, Ahmed MS, Li C, Moreno-Hagelsieb G (2016). The Transporter Classification Database (TCDB): recent advances. Nucleic Acids Res.

[61] Eddy SR (2011). Accelerated profile HMM searches. PLoS Comput Biol.

[62] Yin Y, Mao X, Yang J, Chen X, Mao F, Xu Y (2012). dbCAN: a web resource for automated carbohydrate-active enzyme annotation. Nucleic Acids Res.

[63] Buchfink B, Xie C, Huson DH (2015). Fast and sensitive protein alignment using DIAMOND. Nat Methods.

[64] Lombard V, Golaconda Ramulu H, Drula E, Coutinho PM, Henrissat B (2013). The carbohydrate-active enzymes database (CAZy) in 2013. Nucleic Acids Res.

[65] Tang K, Jiao N, Liu K, Zhang Y, Li S (2012). Distribution and functions of TonB-dependent transporters in marine bacteria and environments: implications for dissolved organic matter utilization. PLoS ONE.

[66] Gómez-Santos N, Glatter T, Koebnik R, Świątek-Połatyńska MA, Søgaard-Andersen L (2019). A TonB-dependent transporter is required for secretion of protease PopC across the bacterial outer membrane. Nat Commun.

[67] Katoh K, Standley DM (2013). MAFFT multiple sequence alignment software version 7: improvements in performance and usability. Mol Biol Evol.

[68] Price MN, Dehal PS, Arkin AP (2010). FastTree 2–approximately maximum-likelihood trees for large alignments. PLoS ONE.

[69] Letunic I, Bork P (2016). Interactive tree of life (iTOL) v3: an online tool for the display and annotation of phylogenetic and other trees. Nucleic Acids Res.

[70] Engel A, Händel N (2011). A novel protocol for determining the concentration and composition of sugars in particulate and in high molecular weight dissolved organic matter (HMW-DOM) in seawater. Mar Chem.

[71] Reintjes G, Fuchs BM, Scharfe M, Wiltshire KH, Amann R, Arnosti C (2020). Short-term changes in polysaccharide utilization mechanisms of marine bacterioplankton during a spring phytoplankton bloom. Environ Microbiol..

[72] Avcı B, Krüger K, Fuchs BM, Teeling H, Amann RI (2020). Polysaccharide niche partitioning of distinct *Polaribacter* clades during North Sea spring algal blooms. ISME J.

[73] Sperling M, Piontek J, Engel A, Wiltshire KH, Niggemann J, Gerdts G (2017). Combined carbohydrates support rich communities of particle-associated marine bacterioplankton. Front Microbiol.

[74] Koch H, Dürwald A, Schweder T, Noriega-Ortega B, Vidal-Melgosa S, Hehemann J-H (2019). Biphasic cellular adaptations and ecological implications of *Alteromonas macleodii* degrading a mixture of algal polysaccharides. ISME J.

[75] Görke B, Stülke J (2008). Carbon catabolite repression in bacteria: many ways to make the most out of nutrients. Nat Rev Microbiol.

[76] Becker S, Scheffel A, Polz MF, Hehemann J-H (2017). Accurate quantification of laminarin in marine organic matter with enzymes from marine microbes. Appl Environ Microbiol.

[77] Becker S, Tebben J, Coffinet S, Wiltshire K, Iversen MH, Harder T (2020). Laminarin is a major molecule in the marine carbon cycle. Proc Natl Acad Sci USA.

