# Supplementary material for: Changing expression patterns of TonB-dependent transporters suggest shifts in polysaccharide consumption over the course of a spring phytoplankton bloom
Source: ISME J. 2021 Mar 1;15(8):2336–50. doi: 10.1038/s41396-021-00928-8 (PMC8319329; doi:10.1038/s41396-021-00928-8)
Supplement: Supplementary file 1 — Supplementary Figure S1 [file 41396_2021_928_MOESM1_ESM.pdf]

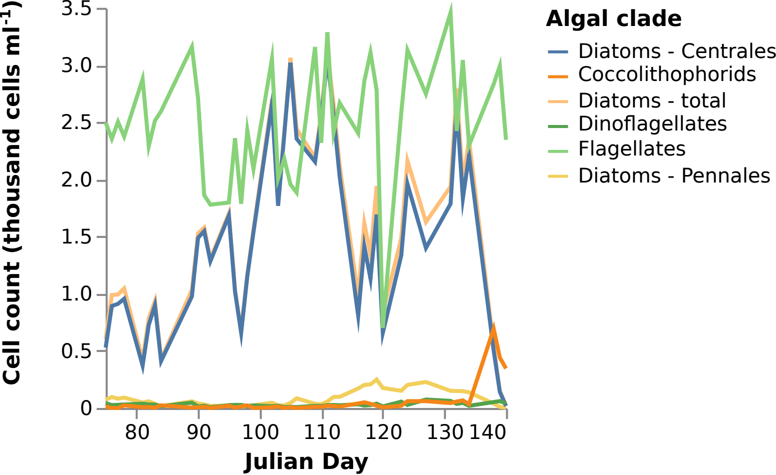

Supplementary figure S1. Cell abundance of different algal clades during the 2016 spring bloom at Helgoland.
