# Supplementary material for: Changing expression patterns of TonB-dependent transporters suggest shifts in polysaccharide consumption over the course of a spring phytoplankton bloom
Source: ISME J. 2021 Mar 1;15(8):2336–50. doi: 10.1038/s41396-021-00928-8 (PMC8319329; doi:10.1038/s41396-021-00928-8)
Supplement: Supplementary file 2 — Supplementary Figure S2 [file 41396_2021_928_MOESM2_ESM.pdf]

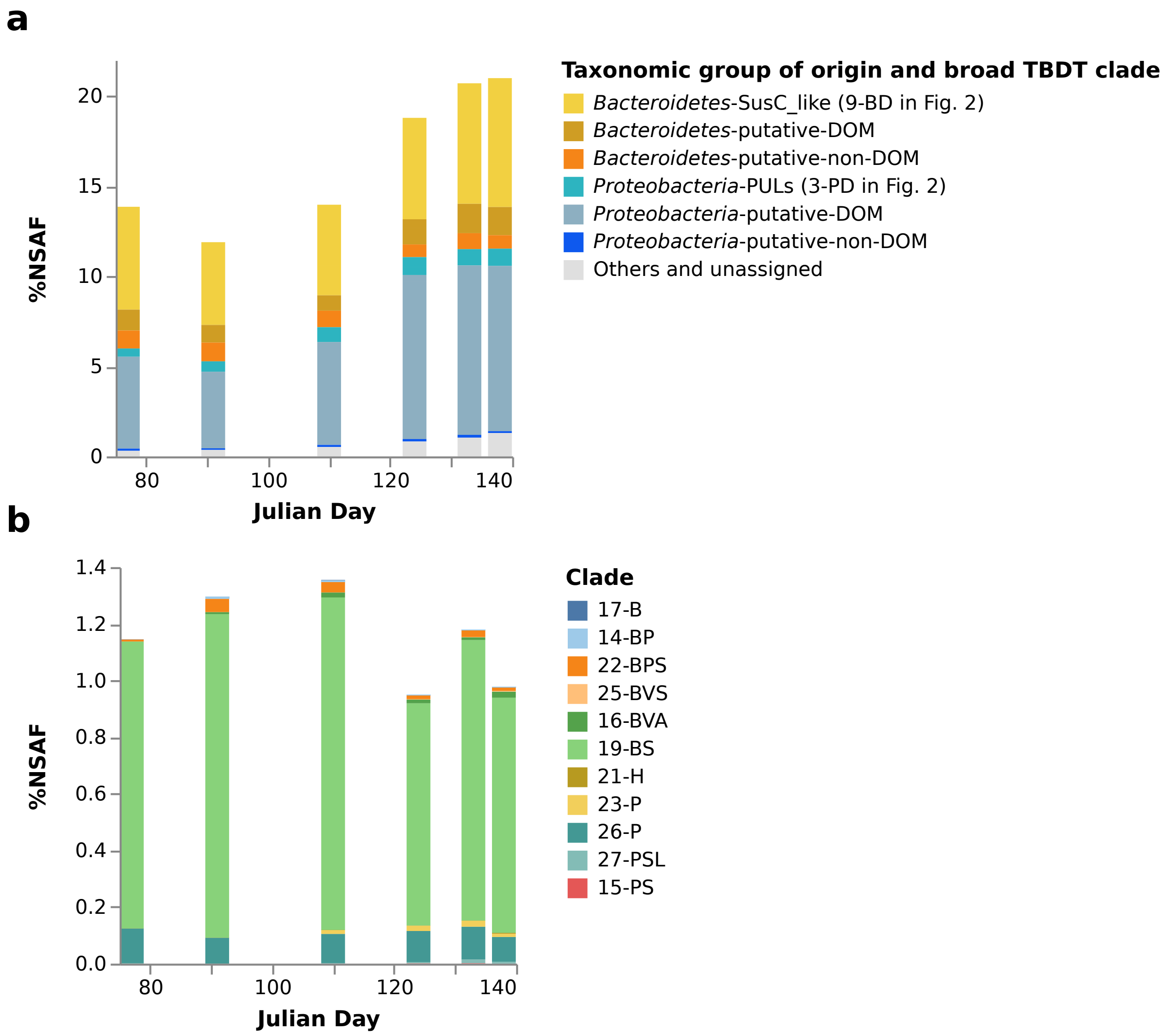

Supplementary figure 2. a) abundance of TBDTs over the course of the 2016 spring phytoplankton bloom, divided by major clade and substrate category. **b)** Protein abundance (%NSAF) of putative non-DOM transporting TBDT. Categories refer to those named in Fig. 2.
