# Supplementary material for: Changing expression patterns of TonB-dependent transporters suggest shifts in polysaccharide consumption over the course of a spring phytoplankton bloom
Source: ISME J. 2021 Mar 1;15(8):2336–50. doi: 10.1038/s41396-021-00928-8 (PMC8319329; doi:10.1038/s41396-021-00928-8)
Supplement: Supplementary file 3 — Supplementary Figure S3 [file 41396_2021_928_MOESM3_ESM.pdf]

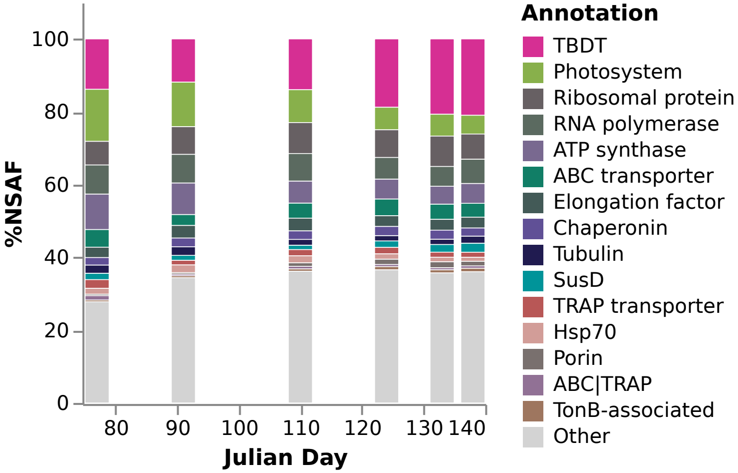

Supplementary figure S3. Protein abundance (%NSAF) across the six metaproteome samples for all detected proteins, grouped by TCDB and Pfam annotations for which average abundance was greater than 1% across the six samples. The category "Photosystem" includes Pfam results for all parts of photosystems I and II, as well as RuBisCO, chlorophyll binding proteins, and phycobilisome proteins, as these categories were covariant. Proteins annotated as both TRAP and ABC transporters are labelled as such. TonB-associated includes the TonB protein, as well as ExbB, ExbD, and TolR annotations.
