# Supplementary material for: Changing expression patterns of TonB-dependent transporters suggest shifts in polysaccharide consumption over the course of a spring phytoplankton bloom
Source: ISME J. 2021 Mar 1;15(8):2336–50. doi: 10.1038/s41396-021-00928-8 (PMC8319329; doi:10.1038/s41396-021-00928-8)
Supplement: Supplementary file 9 — Supplementary text [file 41396_2021_928_MOESM9_ESM.docx]

**Supplementary Text**

**Extended Methods**

*Metagenome sequencing, assembly, and binning*

For assembly for MAG generation, raw reads were quality trimmed using BBduk v35.14 (https://jgi.doe.gov/data-and-tools/bbtools/), with the following parameters ktrim = r, k = 28, mink = 12, hdist = 1, tbo = t, tpe = t, qtrim = rl, trimq = 20, and minlength = 100, while TruSeq adapters were also removed. Reads were assembled using SPAdes v3.10.1 (1) in --meta mode, with kmers 21, 33, 55, 77, and 99, and read error correction enabled. Contigs longer than 2 500 bp were retained for further use.

Binning of assembled metagenomes used all nine error-corrected readsets to generate differential coverage information. For this, reads were mapped using BBmap v35.14 in fast mode, with minid=99 and idfilter=97. Binning followed the standard anvi’o v4 (2) pipeline for metagenomics including initial automated binning with CONCOCT (3), and bin refinement to produce MAGs using the anvi’o interactive interface. Default parameters were used during anvi’o profile creation and merging.

*Metaproteome analyses – sample preparation*

Initially one eighth of a filter was cut into approximately 5 × 5 mm fragments and transferred to low binding tubes (Sorenson BioScience, Salt Lake City, UT, USA) containing 100 µl resuspension buffer 1 (50 mM Tris-HCl (pH 7.5), 0.1 mg ml^-1^ chloramphenicol, 1 mM phenylmethylsulfonyl fluoride (PMSF)). Next these were incubated in 150 µl resuspension buffer 2 (20 mM Tris-HCl pH 7.5, 2% SDS) for 10 min at 60 °C and 1 200 rpm in a thermo-mixer (Eppendorf, Wesseling-Berzdorf, Germany). After addition of 500 µl DNAse buffer (20 mM Tris-HCl pH 7.5, 0.1 mg ml^-1^ MgCl2, 1 mM PMSF, 1 μg ml^-1^ DNAse I) the cells were lysed by ultra-sonication (amplitude 51-60%; cycle 0.5; 4 × 2 min) and kept on ice. The lysate was incubated in the thermo-mixer for 10 min at 37 °C and 1 200 rpm. After centrifugation at 10 000 × g for 10 min at 4 °C the supernatant (protein extract) was collected and the pelleted filter pieces were stirred and centrifuged again at 10 000 × g for 10 min at 4 °C. The supernatant was added to the previously collected supernatant in the same tubes. Proteins were then precipitated by adding pre-cooled trichloroacetic acid (TCA, 20% v/v). After centrifugation (12 000 × g, 30 min, 4 °C), the protein pellets were washed in pre-cooled (‑20 °C) acetone (3 × 10 min,12 000 × g, 4 °C) and dried by vacuum centrifugation at room temperature. Proteins were resuspended in 2× SDS sample buffer (4% SDS (w/v), 20% glycerine (w/v), 100 mM tris-HCl pH 6.8, bromphenol blue (tip of a spatula, to add color), 3.6% 2‑mercaptoethanol (v/v) (freshly added before use)), and separated by 1D PAGE. After separation, each gel lane was cut in either 20 (for the first two replicates) or 10 (for the third replicate) pieces, and trypsin-digested as described previously in ref. (4).

*Metaproteome analyses - LC-MS/MS analysis*

For measurement, an Easy-nLC II (Thermo Fisher Scientific, Waltham, MA, USA) was coupled to an LTQ Orbitrap Velos mass spectrometer (Thermo Fisher Scientific). Samples were loaded onto in-house packed capillary columns of 20 cm or 30 cm length and 100 µm inner diameter. Columns were filled with 3 µm ReproSil-Pur 120 C18-AQ (Dr. Maisch GmbH, Ammerbuch-Entringen, Germany). Peptides were separated using either a 156 min (first two replicates), or 226 min (third replicate) nonlinear binary gradient from 1% to 99% solvent B (95% (v/v) acetonitrile, 0.1% (v/v) acetic acid) in solvent A (0.1% (v/v) acetic acid) at a constant flow rate of 300 nl min^-1^ and 45 °C. The survey scan was carried out with a resolution of R=60k @ m/z 400 followed by CID fragmentation of the 20 most abundant precursor ions (top20). Dynamic exclusion was enabled.

All MS/MS spectra were analysed using Mascot (Matrix Science, London, UK; version 2.6.0). Mascot was set up to search the database containing all protein sequences from the nine metagenomes obtained during the spring bloom 2016 assuming the digestion enzyme trypsin.

For database construction, redundant proteins from the nine metagenomic samples were removed using cd-hit, threshold: 99% identity (5), creating a database with 6 279 079 protein sequence entries. A set of 42 common laboratory contaminants was also added, amounting to 6 279 121 sequences in the final database.

The database search was performed with a fragment ion mass tolerance of 0.50 Da and a parent ion tolerance of 10.0 PPM. Oxidation of methionine was specified as a variable modification. Scaffold v4.8.6 (Proteome Software Inc., Portland, OR) was used to merge the search results and to validate MS/MS based peptide and protein identifications. During creation of the Scaffold file an additional X! Tandem search was performed for validation (The GPM, thegpm.org; version CYCLONE (2010.12.01.1)) with default settings. Peptide identifications were accepted if they could be established at greater than 95.0% probability by the Scaffold Local FDR algorithm. Protein identifications were accepted if they could be established at greater than 99.0% probability and contained at least 2 exclusive unique peptides. Protein probabilities were assigned by the Protein Prophet algorithm (6). Proteins that contained similar peptides and could not be differentiated based on MS/MS analysis alone were grouped to satisfy the principles of parsimony.

For (semi-)quantitative analysis, percent normalised spectral abundance factor (%NSAF) values were calculated based on the number of spectra (total spectral counts) obtained per protein group in each sample (7)*.* Despite the different sample preparation procedure of replicate 1 and 2 compared to 3, these three replicates provided very similar results. Therefore, average values were calculated from three biological replicates (values for proteins that were not identified within a replicate are included as “0” in the calculation).

Mass spectrometry proteomic data have been deposited to the ProteomeXchange Consortium via the PRIDE partner repository (8) and are available through the identifier PXD019294*.*

*Prediction of TBDTs*

TonB dependent transporters were assigned using the PFAM profiles PF00593 (TonB-dependent receptor), PF07715 (TonB-dependent receptor plug domain), PF13620 (Carboxypeptidase regulatory-like domain), and PF13715 (CarboxypepD_reg-like domain); and TIGRFAM profiles TIGR01779 (TonB-dependent vitamin B12 receptor), TIGR01782 (TonB-dependent receptor), TIGR01783 (TonB-dependent siderophore receptor), TIGR01785 (TonB-dependent heme/hemoglobin receptor family protein), TIGR01786 (TonB-dependent hemoglobin/transferrin/lactoferrin receptor family protein), and TIGR04056 (TonB-linked outer membrane protein, SusC/RagA family). TIGR04056 matches specifically the *Bacteroidetes* *susC*-like gene.

*PUL prediction*

Putative laminarin specificity was predicted based on presence of both GH3 and GH16 (9) genes, presence of GH17 (10) genes, or presence of GH30 (10) genes with either GH17 or GH16 genes. Putative alginate specificity was predicted if any combination of PL6 (11), PL7 (12) or PL17 (13) family genes was present. Putative mannose-containing polysaccharide was predicted based on presence of GH92 (14) or GH130 (15) family genes, putative fucose-containing polysaccharide based on presence of GH29 (16) family genes, and putative xylose-containing polysaccharide based on presence of GH11 (17) genes. Finally, putative porphyran specificity was predicted based on presence of both GH29 and GH86 (18) family genes.

**Extended Results**

*SusC-like and TonB dependent transporter protein abundance*

Overall abundance of TBDTs was evenly divided between the *Bacteroidetes* and the *Gammaproteobacteria*, among those with a specific predicted polysaccharide substrate or substrate class, the cumulative %NSAF for *Gammaproteobacteria* (average %NSAF = 0.38, s.d. = 0.11%) was between approximately one-fifth and one-third as large as that for *Bacteroidetes* (average %NSAF = 1.64%, s.d. = 0.16%). Together these TBDTs had average %NSAF of 2.02 (s.d. = 0.18%) (Fig. 3a, b). Average abundance of TBDTs associated with CAZymes without a clearly predictable substrate was 1.45% (s.d. = 0.33%, Fig. 3a, b).

The bulk of the *Bacteroidetes* expressed proteins belonged to the SusC-like clade (mean %NSAF = 6.0, s.d. = 1.1%, Supplementary Fig. S1a). Additionally belonging to the *Bacteroidetes* were proteins in putative DOM-transporting clades that were not SusC-like (average %NSAF: 1.3, s.d. = 0.3%), but which still contained sequences associated with CAZymes (one mannose-containing polymer, one targeting alpha-glucan, and one gene co-located with CAZyme genes targeting an unknown substrate, (Fig. 2)). Among gammaproteobacterial TBDTs, there was division of detected expressed proteins among the multiple sub-clades. Once again, however, the highest overall abundance was seen for members of the groups where few if any reference sequences were present, but where a small handful of the proteins were associated in genomes with laminarin degrading CAZymes or other CAZymes where substrate could not be predicted (collectively *Proteobacteria* putative DOM, Supplementary Fig. S1a, average %NSAF = 7.1). This is substantially greater than that for the clade where membership of PUL-like structures was more common (average %NSAF = 0.8).

*Species-specific expression*

Observing individual species and their gene expression, multiple TBDTs belonging to single species of both *Bacteroidetes* and *Gammaproteobacteria* (maximum 41, mean = 6.46 per species, sd = 6.34) were detected. But only rarely was the most highly abundant TBDT for an individual species one with an assigned polysaccharide substrate (Supplementary Fig. S3). The single highest detection of a TBDT was a SusC-like protein belonging to the species of the genus *Aurantivirga* with representative MAG 20120412_Bin_1_20 (protein Ga0206125_100034462, Supplementary Table S2); this single protein accounted for 0.70% of all detected proteins at its highest point. The highest %NSAF observed for a protein where a polysaccharide substrate was predicted was 0.47% of all proteins. Again this was a SusC-like protein, in this case predicted to transport alpha-glucan, and again belonging to a member of the genus *Aurantivirga* represented by MAG 20110530_Bin_43_1 (Fig. 4, Supplementary Fig. S3).

It is clear that the distribution of TBDTs where substrates could be predicted was relatively even across species, with the *Aurantivirga* species in Supplementary Fig. S3 somewhat unusual in having four TBDTs with a predicted polysaccharide uptake function. That said, it is not unusual for TBDTs where we can predict a polysaccharide substrate to be among the more abundant proteins (Fig. 4, Supplementary Fig. S3).

The four genus level clades with the highest overall %NSAF values for TBDTs are represented by individual exemplar species in Supplementary Fig. S3. The genus with the highest total abundance of TBDTs across the whole dataset is *Aurantivirga* (Genome Tree Database (GTDB) classification: SCGC-AAA160-P02), which accounted for, between its four species, 13.3% of all TBDT NSAF (max. = 29.2%, min. = 6.4%). Following this were the two gammaproteobacterial genera SAR92 (GTDB: HTCC2207) and *Luminiphilus*, accounting for 10.5% and 8.0% respectively, and then the one *Bacteroidetes* genus belonging to the NS4 clade (GTDB: MAG-121220-bin8) – accounting for 7.8% of all TBDT NSAF. These clades were also among the most important PUL containing clades (e.g. Fig. 4).

**Supplementary references**

1. Nurk S, Meleshko D, Korobeynikov A, Pevzner PA. metaSPAdes: a new versatile metagenomic assembler. Genome Res. 2017;27:824–34.

2. Eren AM, Esen ÖC, Quince C, Vineis JH, Morrison HG, Sogin ML, et al. Anvi’o: an advanced analysis and visualization platform for ‘omics data. PeerJ. 2015;3:e1319.

3. Alneberg J, Bjarnason BS, de Bruijn I, Schirmer M, Quick J, Ijaz UZ, et al. Binning metagenomic contigs by coverage and composition. Nat Methods. 2014;11:1144–6.

4. Teeling H, Fuchs BM, Becher D, Klockow C, Gardebrecht A, Bennke CM, et al. Substrate-controlled succession of marine bacterioplankton populations induced by a phytoplankton bloom. Science. 2012;336:608–11.

5. Li W, Godzik A. Cd-hit: a fast program for clustering and comparing large sets of protein or nucleotide sequences. Bioinformatics. 2006;22:1658–9.

6. Nesvizhskii AI, Keller A, Kolker E, Aebersold R. A statistical model for identifying proteins by tandem mass spectrometry. Anal Chem. 2003;75:4646–58.

7. Florens L, Carozza MJ, Swanson SK, Fournier M, Coleman MK, Workman JL, et al. Analyzing chromatin remodeling complexes using shotgun proteomics and normalized spectral abundance factors. Methods San Diego Calif. 2006;40:303–11.

8. Perez-Riverol Y, Csordas A, Bai J, Bernal-Llinares M, Hewapathirana S, Kundu DJ, et al. The PRIDE database and related tools and resources in 2019: improving support for quantification data. Nucleic Acids Res. 2019;47:D442–50.

9. Labourel A, Jam M, Jeudy A, Hehemann J-H, Czjzek M, Michel G. The β-glucanase ZgLamA from *Zobellia galactanivorans* evolved a bent active site adapted for efficient degradation of algal laminarin. J Biol Chem. 2014;289:2027–42.

10. Becker S, Scheffel A, Polz MF, Hehemann J-H. Accurate quantification of laminarin in marine organic matter with enzymes from marine microbes. Vieille C, editor. Appl Environ Microbiol. 2017;83:e03389-16.

11. Mathieu S, Henrissat B, Labre F, Skjåk-Bræk G, Helbert W. Functional exploration of the polysaccharide lyase family PL6. PLoS ONE. 2016;11:e0159415.

12. Thomas F, Lundqvist LCE, Jam M, Jeudy A, Barbeyron T, Sandström C, et al. Comparative characterization of two marine alginate lyases from *Zobellia* *galactanivorans* reveals distinct modes of action and exquisite adaptation to their natural substrate. J Biol Chem. 2013;288:23021–37.

13. Park D, Jagtap S, Nair SK. Structure of a PL17 family alginate lyase demonstrates functional similarities among exotype depolymerases. J Biol Chem. 2014;289:8645–55.

14. Zhu Y, Suits MDL, Thompson AJ, Chavan S, Dinev Z, Dumon C, et al. Mechanistic insights into a Ca 2+ -dependent family of α-mannosidases in a human gut symbiont. Nat Chem Biol. 2010;6:125–32.

15. Cuskin F, Baslé A, Ladevèze S, Day AM, Gilbert HJ, Davies GJ, et al. The GH130 family of mannoside phosphorylases contains glycoside hydrolases that target β-1,2-mannosidic linkages in *Candida* mannan. J Biol Chem. 2015;290:25023–33.

16. Sulzenbacher G, Bignon C, Nishimura T, Tarling CA, Withers SG, Henrissat B, et al. Crystal Structure of Thermotoga maritima α-l-Fucosidase Insights into the catalytic mechanism and the molecular basis for fucosidosis. J Biol Chem. 2004;279:13119–28.

17. Paës G, Berrin J-G, Beaugrand J. GH11 xylanases: Structure/function/properties relationships and applications. Biotechnol Adv. 2012;30:564–92.

18. Hehemann J-H, Kelly AG, Pudlo NA, Martens EC, Boraston AB. Bacteria of the human gut microbiome catabolize red seaweed glycans with carbohydrate-active enzyme updates from extrinsic microbes. Proc Natl Acad Sci. 2012;109:19786–91.
