# Supplementary material for: Changing expression patterns of TonB-dependent transporters suggest shifts in polysaccharide consumption over the course of a spring phytoplankton bloom
Source: ISME J. 2021 Mar 1;15(8):2336–50. doi: 10.1038/s41396-021-00928-8 (PMC8319329; doi:10.1038/s41396-021-00928-8)
Supplement: Supplementary file 4 — Supplementary Figure S4 [file 41396_2021_928_MOESM4_ESM.pdf]

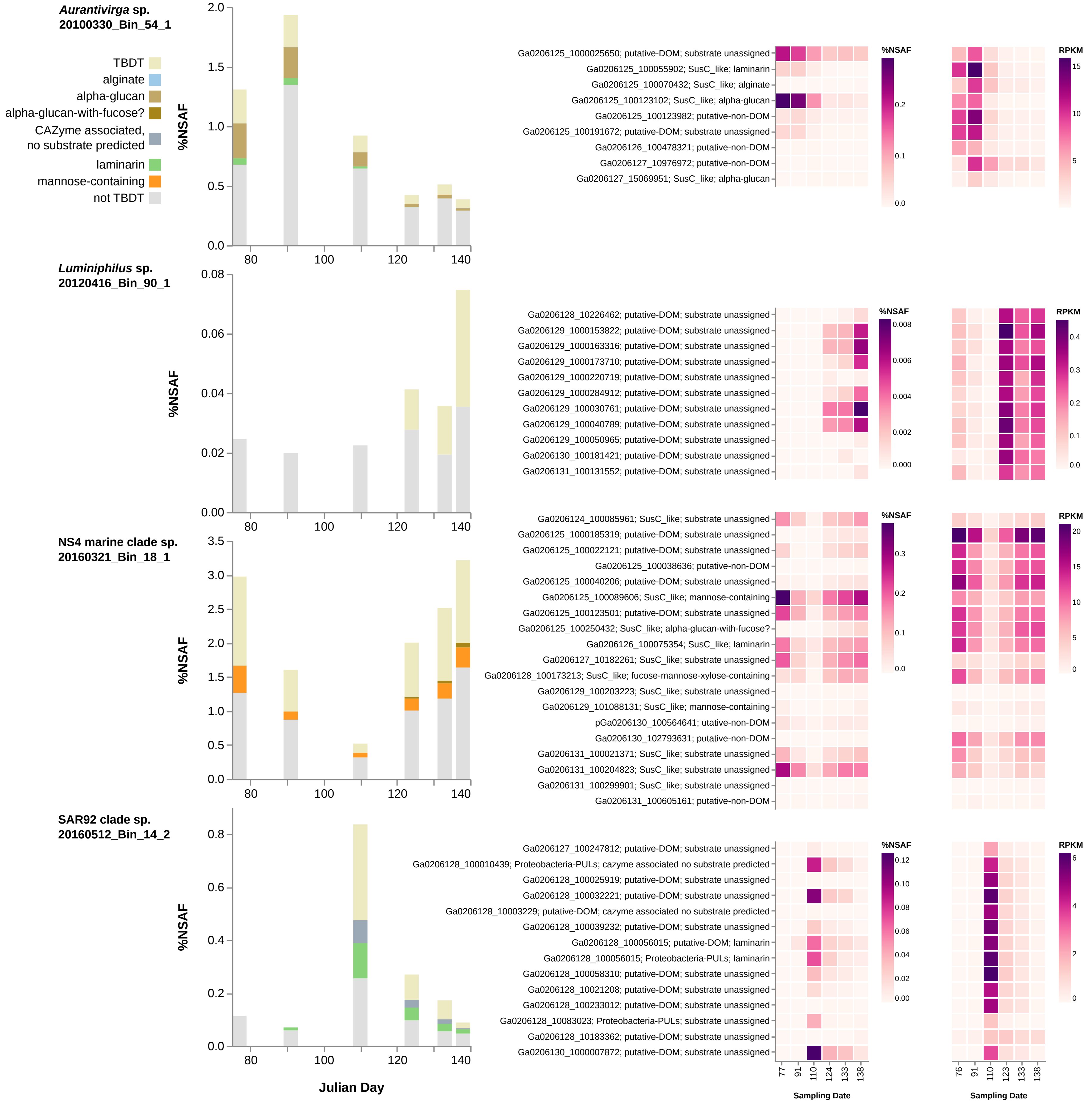

Supplementary figure 4. Protein abundance in exemplar species from the four clades with the highest overall protein abundance. Barplots show abundance of all proteins, while heatmaps indicate protein abundance and gene frequency for all detected TBDTs of that species, including strain specific genes (evidenced by lower RPKM values).
